# The Anti-*Phytophthora* Effect of Selected Potato-Associated *Pseudomonas* Strains: From the Laboratory to the Field

**DOI:** 10.3389/fmicb.2015.01309

**Published:** 2015-11-27

**Authors:** Anouk Guyer, Mout De Vrieze, Denise Bönisch, Ramona Gloor, Tomke Musa, Natacha Bodenhausen, Aurélien Bailly, Laure Weisskopf

**Affiliations:** ^1^Agroscope, Institute of Sustainability SciencesZurich, Switzerland; ^2^Agroscope, Institute of Plant Production SciencesNyon, Switzerland; ^3^Viticulture and Oenology, CHANGINS, University of Applied Sciences and Arts Western SwitzerlandNyon, Switzerland

**Keywords:** *Phytophthora*, *Pseudomonas*, *Solanum tuberosum*, leaf disk, biocontrol

## Abstract

Late blight, caused by the oomycete *Phytophthora infestans*, is the most devastating disease of potato. In organic farming, late blight is controlled by repeated applications of copper-based products, which negatively impact the environment. To find alternative solutions for late blight management, we have previously isolated a large collection of bacteria from the phyllosphere and the rhizosphere of potatoes. Here we report the antagonistic potential of these strains when co-cultivated with *P. infestans* as well as with other potato pathogens. We then focused on three *Pseudomonas* strains and compared their protective impact against late blight to that of well-known biocontrol strains *in planta* using a high-throughput leaf disk assay with automated picture analysis. When sprayed on the leaves of potatoes in the greenhouse, the strains were able to survive for at least 15 days. Under field conditions, populations decreased faster but all tested strains could still be retrieved after 8 days. The most active strain *in vitro*, *P. chlororaphis* R47, was also the best protectant on leaf disks from plants grown in the greenhouse experiment, but its protection potential could not be verified in the field due to unfavorable infection conditions. However, its protective effect against *P. infestans in planta*, its survival in the phyllosphere as well as its ability to colonize the potato rhizosphere in very high population densities, suggest a potential for field application, e.g., in the form of tuber treatment or leaf spray.

## Introduction

Over the last decades, the need to move from intensive agriculture to a more sustainable way of food production has risen in the awareness of growers and consumers. However, crop production is threatened by a variety of abiotic and biotic factors, such as changing climate or the occurrence of disease-causing agents. In potato production, yield losses are mostly due to the oomycete *Phytophthora infestans*, which causes late blight and can lead, upon favorable infection conditions, to massive destruction of the crop within a few days (Fry, 2008). In conventional farming, late blight is typically controlled through repeated applications of various fungicides, whereas copper-based products are commonly used in organic farming (Cooke et al., 2011; Axel et al., 2012). In view of its accumulation in the soil and of its toxicity toward the soil fauna, copper use represents an environmental hazard and alternative solutions to control late blight are needed to ensure sustainable potato production (Dorn et al., 2007). Biocontrol organisms have been suggested as a putative alternative to chemical protectants in the protection against diseases (Velivelli et al., 2014). Such antagonistic bacteria or fungi have been proven efficient under field conditions and some of them are available as commercial products and routinely used by farmers, such as *Pseudomonas chlororaphis* MA 342, which protects cereals against some seed-borne fungal diseases (Johnsson et al., 1998), or *Bacillus amyloliquefaciens* FZB42, which acts both as a plant growth promoting and as a biocontrol agent (Chen et al., 2007). However, although numerous studies have tested the effect of microbial inoculants on late blight (reviewed in Axel et al., 2012), none has so far demonstrated a protection against late blight in the field. This might be at least partly due to the fast spreading of *Phytophthora* during humid conditions, which is mediated by the production of sporangia that, depending on temperature, can either directly infect plant tissues or release motile zoospores, which in turn infect new leaves (Fry, 2008). A successful biocontrol agent would therefore need to be able to inhibit not only the pathogen’s mycelial growth but also the formation and/or the germination of its sporangia, as was recently reported for a *Lysobacter* strain producing cyclic dipeptides (Puopolo et al., 2014). In an attempt to find such a biocontrol agent against potato late blight, we have previously isolated bacterial strains from the phyllosphere and rhizosphere of potato, which we hypothesized to be adapted to the host plant (Hunziker et al., 2015). In this earlier work, we reported the ability of potato-associated bacteria to inhibit growth and sporulation of *Phytophthora infestans* through the emission of volatile organic compounds (Hunziker et al., 2015). However, the gap between results obtained in controlled laboratory conditions and true protective potential in the field can be very large (Dorn et al., 2007) and further studies are needed that include testing the strains’ ability to establish in sufficient densities in the targeted plant organs (roots vs. shoots) and their efficiency *in planta* as well as under field conditions. The present study investigates these questions using a subset of *Pseudomonas* strains which showed promising protective effects *in vitro.* The strains’ root and leaf colonization capacity as well as their effect on plant growth and development were assessed. Their protective effect *in planta* was then analyzed using a newly developed high through-put leaf disk assay. Finally, the strains’ survival and efficiency under field conditions was monitored in a microplot experiment.

## Materials and Methods

### Chemicals and Culture Media

Luria-Bertani medium (LB) was used to cultivate bacteria. LB agar plates were prepared by dissolving 20 g L^-1^ of Difco LB Broth, Lennox (BD) mixed with 15 g L^-1^ agar (Agar Agar, ERNE surface AG). PIA medium was prepared by dissolving 45 g L^-1^ of *Pseudomonas* Isolation Agar (Fluka) in distilled water to which 20 ml L^-1^ of glycerol (Sigma–Aldrich) was added. Fungi and oomycetes grew on rye agar (RA), malt agar (MA), or potato dextrose agar (PDA). RA was prepared by simmering 200 g rye grains (winter rye cv. Picasso95) in tap water for ca. 1 h. The filtered liquid (1.5 mm mesh) was filled up to a volume of 1 L with tap water and 20 g L^-1^ agar was added. For the initial screen (**Table 1**), RA without glucose was used, for later experiments, RA was supplemented with 5 g L^-1^ glucose. MA was prepared with 15 g L^-1^ Difco malt extract agar (BD) and 12 g L^-1^ agar, and PDA contained 39 g L^-1^ PDA (Oxoid). One experiment was performed on water agar (WA) containing dH_2_O and 6 g L^-1^ agar. When needed, rifampicin (PanReac AppliChem) was added at a concentration of 50 μg mL^-1^.

**Table 1:** Antagonistic activity of 32 bacterial strains isolated from rhizosphere (R) or shoots (S) of field-grown potato plants.

Strain	Affiliation	*P. inf*	*H. sol*	*R. sol*	*F. oxy*	*D. dian*
R47	*P. chlororaphis*	**8**	**11**	**69**	**81**	**35**
R32	*P. vranovensis*	**10**	**14**	**51**	**88**	**41**
R84	*P. marginalis*	**22**	**36**	**81**	**92**	**38**
R82	*P. marginalis*	**15**	**12**	**91**	**82**	**73**
S49	*P. fluorescens*	**46**	**37**	**73**	**79**	**51**
R73	*Bacillus* sp.	**7**	**27**	94	**84**	88
R54	*Bacillus* sp.	**13**	**18**	98	**83**	**87**
S50	*P. moraviensis*	**10**	66	**76**	**91**	**58**
S01	*Streptomyces* sp.	**12**	**60**	104	**60**	**81**
R01	*P. moraviensis*	**30**	**66**	**95**	98	**33**
R76	*P. fluorescens*	**25**	**38**	**91**	**93**	**81**
S35	*P. marginalis*	**27**	**67**	**81**	**80**	**73**
S06	*P. frederiksbergensis*	101	**21**	**82**	99	**66**
S24	*P. frederiksbergensis*	**92**	**22**	**95**	**85**	**75**
S22	*P. syringae*	**85**	**22**	**81**	**95**	89
S19	*P. frederiksbergensis*	100	**21**	**89**	**91**	**72**
R95	*P. lini*	72	**21**	**93**	99	97
R02	*P. veronii*	**56**	**60**	**88**	**91**	92
S46	*Curtobacterium* sp.	**35**	**78**	95	**84**	97
S27	*Arthrobacter* sp.	**50**	nd	nd	**90**	94
R74	*P. frederiksbergensis*	**89**	**28**	101	**92**	**83**
R31	*Sporosarcina* sp.	**39**	nd	nd	98	99
S34	*P. jessenii*	**96**	**30**	**90**	99	**81**
R85	*Rhodococcus* sp.	**41**	84	104	**91**	**79**
R29	*Bacillus* sp.	**65**	**53**	100	**94**	**87**
S04	*P. frederiksbergensis*	**97**	**22**	97	98	91
R61	*Arthrobacter* sp.	**8**	113	**115**	99	92
S25	*Curtobacterium* sp.	77	**71**	**90**	98	93
R60	*Arthrobacter* sp.	**15**	**132**	**112**	**87**	93
R75	*P. frederiksbergensis*	82	095	98	**86**	88
R42	*Microbacterium* sp.	**14**	**153**	**107**	**92**	**89**
R96	*Flavobacterium* sp.	111	96	99	**93**	**80**


*Strains were tested against the oomycete *Phytophthora infestans* (*P. inf*), the fungi *Helminthosporium solani* (*H. sol*), *Rhizoctonia solani* (*R. sol*), and *Fusarium oxysporum* (*F. oxy*) as well as against the phytopathogenic bacterium *Dickeya dianthicola* (*D. dian*). Strains are ordered according to their overall activity (most active first). Numbers indicate the average growth of 3–4 replicates in percentage of the control (inhibition in red, stimulation in green). Bold values indicate values significantly different from the control according to a Student’s *t-*test. nd, not determined (strains that did not grow on the fungal medium used in the direct assay).*

### Strains, Culture Conditions and Preparation of Inoculation Suspensions

The bacterial strains used in this study are described in a previous publication (Hunziker et al., 2015). Additionally, *Pseudomonas protegens* CHA0 and *Pseudomonas* DSMZ 13134 were included as controls in most experiments. *Dickeya dianthicola* was obtained from S. Schaerer (Agroscope). For the greenhouse and field experiments, rifampicin-resistant derivatives of selected strains were obtained by streaking a high density of pure bacterial culture onto a LB plate containing rifampicin (50 μg mL^-1^). Spontaneous rifampicin resistant colonies were visible 2 days later; one colony for each strain was streaked on a fresh LB plate containing the same concentration of rifampicin. After 2 days, glycerol stock was prepared with each stable mutant strain. Bacterial strains were kept at -80°C in 25% glycerol for long-term storage. A polyspore isolate of *Phytophthora infestans* sampled in 2001 in Zurich Affoltern (provided by H. Krebs, Agroscope) was used for all experiments. This isolate was grown on RA in the dark at 18°C, and was regularly transferred to potato tuber slices for host passages. The fungi *Rhizoctonia solani*, *Helminthosporium solani*, obtained from P. Frei (Agroscope), were grown on PDA and MA respectively. *Fusarium oxysporum* was recovered from a contaminated *P. infestans* host passage in 2013 and was grown on RA. Long-time storage of all fungi was done in 25% glycerol at -80°C. All potato pathogens were continuously grown on agar media and agar plugs (ø 5 mm) were transferred to fresh medium plates when borders of the previous plate were reached. Plates were stored in the dark at ca 20°C. To obtain *P. infestans* sporangia suspensions, mycelium was detached from overgrown agar plates and suspended in tap water. The suspension was filtered through autoclaved gauze and the number of sporangia adjusted to the desired final concentration using a Thoma chamber (Marienfeld Superior, Germany). The suspension was stored at 5°C in the dark until use. Unless otherwise specified, bacterial suspensions were prepared by harvesting overnight LB agar cultures and resuspending the cells in 0.9% NaCl. For the microplot field application, densities were adjusted by adding tap water.

### *In Vitro* Screening of Bacterial Strains for Activity Against Different Potato Pathogens

Thirty two bacterial strains showing antagonistic potential in a preliminary screening (Hunziker et al., 2015) were tested in a dual culture assay for inhibitory effects toward five different potato pathogens: the oomycete *P. infestans*, the fungi *H. solani*, *R. solani*, *F. oxysporum* and the bacterium *D. dianthicola*. The antagonistic bacteria and the pathogens were grown on the same Petri dish, which was filled with either RA (*P. infestans, F. oxysporum*), PDA (*H. solani, R. solani*), or LB (*D. dianthicola*). Two strains, S27 and R31, could not be grown on PDA and were thus not tested against *H. solani* and *R. solani.* The time point of the application of bacterial strains varied, as the potato pathogens showed different growth speeds. It was on the same day for *D. dianthicola*, 1 day later for *R. solani* and *F. oxysporum*, 4 days later for *P. infestans* and 7 days later for *H. solani*. Each fungus or oomycete was inoculated as a plug and *D. dianthicola* was inoculated as 10 μl drop of liquid culture adjusted to OD_570_ = 1 in the center of the Petri dish. Three 10 μl drops of overnight bacteria cultures adjusted to OD_570_ = 1 (or LB for the control) were spotted at the border of the Petri dishes. Plates were incubated at 20°C in the dark and the pathogen growth area was assessed by picture analysis (after 3 days for *R. solani*, 7 days for *D. dianthicola* and *F. oxysporum*, 14 days for *P. infestans* and 28 days for *H. solani*) using the image processing program ImageJ (Schneider et al., 2012). This experiment was carried out in four replicates per bacterial strain (five control plates). The average growth of the pathogens in presence of the different strains was compared with that of the pathogens grown in absence of the strains (control) and a percentage of control growth was calculated.

### Greenhouse Experiment

Rifampicin-resistant derivatives of selected *Pseudomonas* strains (R47, R76, S35, CHA0, *Pseudomonas* DSMZ 13134, see section above for the generation of these strains) were inoculated onto potato tuber sprouts and tested for their effect on plant development (cv. Charlotte and cv. Victoria) and for their survival in the rhizosphere. For the sprout inoculation experiment, bacterial cells were suspended in 0.9% NaCl and adjusted to OD_570_ = 1. The sprouts were moistened by spraying them with a solution containing 0.9% NaCl and subsequently 1.5 mL of the bacterial cell suspension was pipetted next to the sprout. To prevent a wash out of the inoculated cells, pots were not watered during the first 24 h following inoculation. Additionally bacteria suspensions of the same strains were sprayed onto potato leaves (∼15 mL per plant), in order to assess the survival of the bacterial population for a period of 15 days. Inoculation of potato sprouts was done 1 day before potting, by the application of 1.5 mL unwashed bacterial cells suspended in 0.9% NaCl and adjusted to OD_570_ = 1 (0.9% NaCl was used for the control). Each treatment was replicated six times. BBCH stage and plant height (distance between the soil surface and the apical meristem) were measured once a week between the period from sprouting until flowering (4–33 days after planting). The survival of the sprout-inoculated *Pseudomonas* strains in the rhizosphere was assessed 11 weeks after potting. To this end, the washed root system of two replicate plants was cut in small pieces and suspended in 15 mL 0.9% NaCl. After a sonication step of 5 min, the suspension was tenfold serially diluted and 100 μL of each dilution was plated on a PIA plate supplemented with rifampicin (50 μg L^-1^). After 3 days of incubation at 20°C in the dark, colony forming units (CFUs) were counted from the most appropriate dilution. The survival of bacteria sprayed on potato leaves (OD_570_ = 1) was investigated within a period of 2 weeks at the days 1, 3, 8, and 15. From each treatment three 5 mm diameter leaf disks were cut and suspended in 200 μL 0.9% NaCl. The plant tissue was homogenized using a small plastic pestle and after sonication and tenfold serial dilution (see above), 5 μL of each dilution was spotted twice on a LB plate containing rifampicin, which was then lifted to let the drop fall and spread the CFUs. After 3 days of incubation at 20°C, CFUs were counted from the most appropriate dilution.

### Establishment of a High Through-put Potato Leaf Disk Assay with Automated Picture Analysis to Monitor *P. infestans* Infection

In order to determine the appropriate sporangia concentration as well as the application strategy aiming at optimum infection pressure (necrosis development and sporangiophore appearance on the leaf surface), sporangia suspensions in different concentrations (6.25^.^10^4^, 1.25^.^10^5^, 2.5^.^10^5^, 5^.^10^5^ sporangia mL^-1^) were applied in a 10 μL drop on the upper or the lower side of potato leaf disks (ø 17 mm), cut from potato plants cv. Victoria 39 days after planting. Leaf disks were placed on a previously watered filter paper in a standard Petri dish and inoculated with the respective sporangia suspension. The Petri dishes were placed in a lightproof plastic box and incubated at 18°C for a period of 8 days. When the leaf disks showed first infection symptoms (after 3 days), daily pictures (dimensions 5184 × 3456) were taken with a reflex camera (Canon EOS 700D) and the increase of necrotic plant tissue (days 3–7) and sporangiophore cover (day 8) was analyzed by the automated picture analysis macroinstructions developed for this purpose in the freeware program ImageJ (see Supplementary Material).

### Testing the Protective Potential of Bacteria Applied on Leaf Disk Against *P. infestans*

Using this newly developed leaf disk method with automated picture analysis, the effect of selected bacterial strains (*Pseudomonas* strains R47, R76, S35, CHA0, DSMZ 13134) on disease progression was monitored. To this end, bacteria and sporangia suspensions were mixed and applied on the lower side of leaf disks (cv. Victoria, 18 replicates). The final sporangia concentration was 1.25^.^10^5^ mL^-1^ and bacteria were applied at two population densities: OD_570_ = 0.3 and 3 (corresponding approximately to 2^.^10^8^ and 2^.^10^7^ cells/ml). The experimental set up was the same as described above and after the application of 10 μL of the combined suspensions, the leaf disks were incubated for 8 days at 18°C. The necrotic leaf tissue and the sporangiophores were measured with the automated picture analysis macroinstructions (see Supplementary Material) 4 days after inoculation and 8 days after inoculation respectively. A separate set of leaf disks inoculated with the mixed suspension was used to take microscope pictures of the sporangia, which were exposed to the bacteria at a concentration of OD_570_ = 3 (corresponding approximately to 2^.^10^9^ cells/ml). Pictures were taken 4 days after inoculation, when the necrotic area of the control treatment reached 60% of the leaf disk area.

### Sporangia Germination

The sporangia germination in mixed sporangia-bacteria suspension was analyzed, when sporangia were exposed to the strains in population densities of OD_570_ = 3, OD_570_ = 0.3 and OD_570_ = 0.03 (0.9% NaCl was used as control). Fifteen micro liter of the mixed suspension was applied on a 0.6% WA plug placed on microscope glass slides. The sporangia germination behavior was assessed after 3 days of incubation at 18°C in the dark. Treatments and controls were replicated four times and randomly placed on the glass slides. Additional control plugs (*n* = 20) were incubated separately from treated plugs to verify whether the control plugs incubated on the same glass slides as the treated ones would be influenced in any way. The number of germinating sporangia per plug was calculated as percent of germinated sporangia relative to the total number of sporangia (23–106 per plug depending on sporangia density). This percentage was then compared to the germination percentage of the control.

### Microplot Experiment

In order to determine the protection potential of bacterial strains under field conditions, a microplot experiment was carried out in Zurich Affoltern, Switzerland. Each microplot consisted of one row of five potato plants. Per treatment, four replicates were allocated to four blocks in which they were randomly distributed. Each block was surrounded on all sides by a single row of border plants of the cultivar Panda (low susceptibility to late blight). The blocks with borders were separated from one another by a single row of plants of the cultivar Bintje (high susceptibility to late blight). The plants (cv. Victoria) were planted on 15th of April 2015. Distances were 30 cm between plants and 70 cm between rows. From day 44 after planting on, the plants were regularly sprayed with bacterial suspensions of the selected strains (R47, R76, S35, CHA0 and *Pseudomonas* DSMZ 13134), according to the recommendations of the late blight decision support system Bio-PhytoPRE (Musa-Steenblock and Forrer, 2005). The treatment interval ranged between 6 days and 2 weeks. In total, six treatments were carried out. For the suspensions, overnight bacterial cultures were suspended in water and supplemented with 0.1% Nu-Film 17^®^ (Andermatt Biocontrol), a wetting agent intended to enhance adhesion of the cells to the leaf surface. The concentration of the suspensions was adjusted to OD_570_ = 1. The suspensions were sprayed from above and below on the plants’ leaves, each plant receiving approximately 50 mL of suspension per application. After the last application, which occurred 106 days after planting, the survival of sprayed bacteria was assessed one and 8 days after this last spraying. To this end, three leaf discs (ø 17 mm) were cut and suspended in 2 mL 0.9% NaCl. The leaf tissue was further homogenized using a Polytron PT300 homogeniser (Kinematica AG), with which the samples were shredded by 6000 rpm during approximately 30 s each. After 5 min in a sonication bath, samples were tenfold serially diluted. Five micro liter of each dilution was spotted twice on a PIA plate containing rifampicin and incubated at 20°C in the dark. CFUs were counted from the most appropriate dilution after 3 days incubation. In order to assess the protective potential of the strains, a leaf disk experiment was performed 1 day after spraying as described above. Sporangiophore cover was assessed 6 days after inoculation of the leaf disk.

### Statistical Evaluation

In the initial screen (**Table 1**), a Student’s *t*-test was used to compare the growth inhibition of each bacterial strain to the negative control. The evaluation of subsequent experiments was done with GraphPad Prism version 5.01 (GraphPad Software, San Diego, CA, USA), performing one-way ANOVAs with Tukey’s *post hoc* tests.

## Results

### Growth Inhibition of Pathogens by Potato-associated Bacterial Strains in Dual Cultures

Thirty two potato-associated bacterial strains previously identified as potentially active based on their emission of volatiles (Hunziker et al., 2015) were tested for their effects in direct competition assays with five different potato pathogens: the oomycete *Phytophthora infestans*, the fungi *Helminthosporium solani*, *Rhizoctonia solani*, *Fusarium oxysporum* and the bacterium *Dickeya dianthicola*. Among those 32 strains, five *Pseudomonas* were able to significantly inhibit the growth of each target organism, although *R. solani* and *F. oxysporum* were inhibited to a much lesser extent than *P. infestans* and *H. solani* (**Table 1**). This differential reaction of the target organisms to the bacterial strains was further illustrated, e.g., by the fact that *R. solani* was more inhibited in its growth by *Pseudomonas* strains than by the other strains, while *Bacillus strains* R73 and R54 were able to drastically reduce the growth of both *P. infestans* and *H. solani* and the *Streptomyces* strain S01 was the best inhibitor of *F. oxysporum* (60% of its control growth) (**Table 1**). Within the genus *Pseudomonas*, which in general inhibited *P. infestans* more strongly than other targets, the strains affiliated *P. frederiksbergensis* (e.g., S04, S06, S19, S24, R74) all impacted *H. solani* more than *P. infestans*. In contrast, the two *Arthrobacter* strains R61 and R60 drastically reduced the growth of *P. infestans* but barely affected the other target organisms. Based on these results, we selected three promising strains, which showed significant *in vitro* growth inhibition of all pathogens, for further analysis: *P. chlororaphis* R47, the strain with the highest *in vitro* inhibition of *P. infestans*, as well as *P. fluorescens* R76 and *P. marginalis* S35, which showed comparable *in vitro* inhibition but were isolated from different plant parts (rhizosphere for R76 and phyllosphere for S35). In the following experiments, two control *Pseudomonas* strains were included for comparison purposes, *P. protegens* CHA0 (Voisard et al., 1989) and *Pseudomonas* sp. DSMZ 13134.

### Effects of Sprout-inoculated Bacterial Strains on Growth and Development of Potato Plants

**FIGURE 1:**
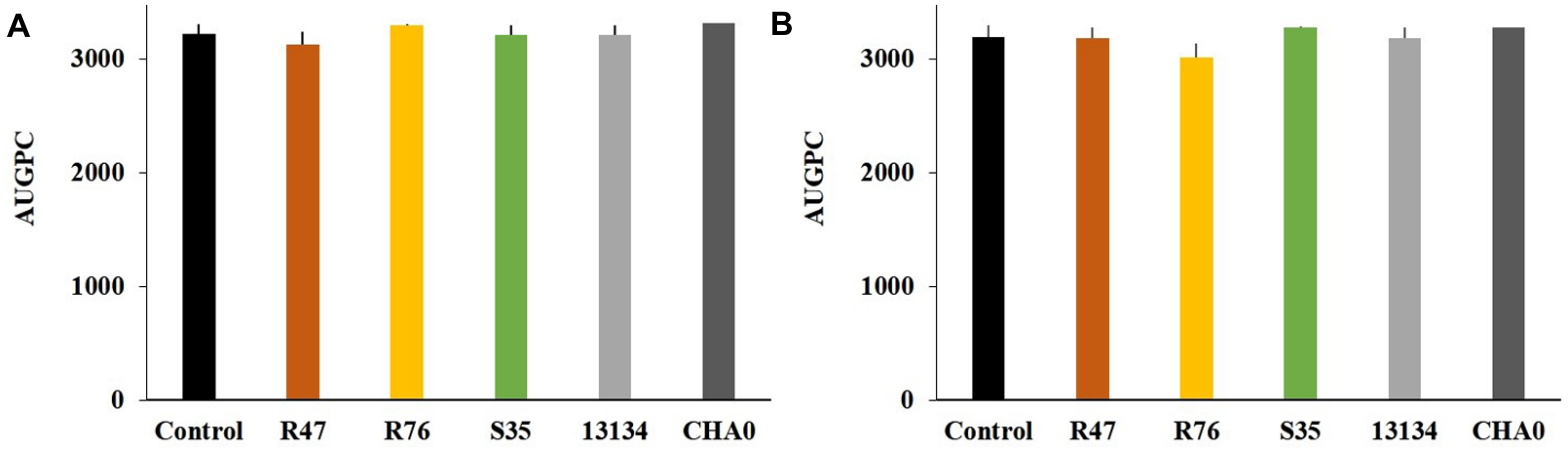
**Effects of sprout-inoculated bacteria on the growth of the potato varieties Charlotte (A) and Victoria (B).** Overall growth was calculated as AUGPC (the area under growth progression curve, d(BBCH)/dt) (see **Supplementary Figure S1**). Averages and standard errors are shown. Sprout inoculation did not result in any significant change in the growth of cv. Charlotte [*F*(5) = 1.36, *P* = 0.27] or cv. Victoria [*F*(5) = 0.74, *P* = 0.60] as determined by one-way ANOVA.

As a first step toward evaluating the potential of these three selected strains for practical application, it was assessed whether they showed any phytotoxic effect when applied onto the potato tubers. The bacterial strains were inoculated on the tuber sprouts of two different potato cultivars, Victoria (moderately susceptible to late blight) and Charlotte (highly susceptible to late blight), which were monitored for 33 days after planting (**Supplementary Figure S1**). No significant difference in overall growth between the plants developing from inoculated and non-inoculated sprouts could be observed, thus excluding a phytotoxic effect of the strains (**Figure 1**). No growth promotion was observed either, but some strains led to a more constant growth, i.e., to less variability between individual plants, for instance *P. fluorescens* R76 in the cultivar Charlotte and *P. protegens* CHA0 in both cultivars (**Figure 1**).

### Survival of the Strains in the Phyllosphere and Rhizosphere of Potato Plants

**FIGURE 2:**
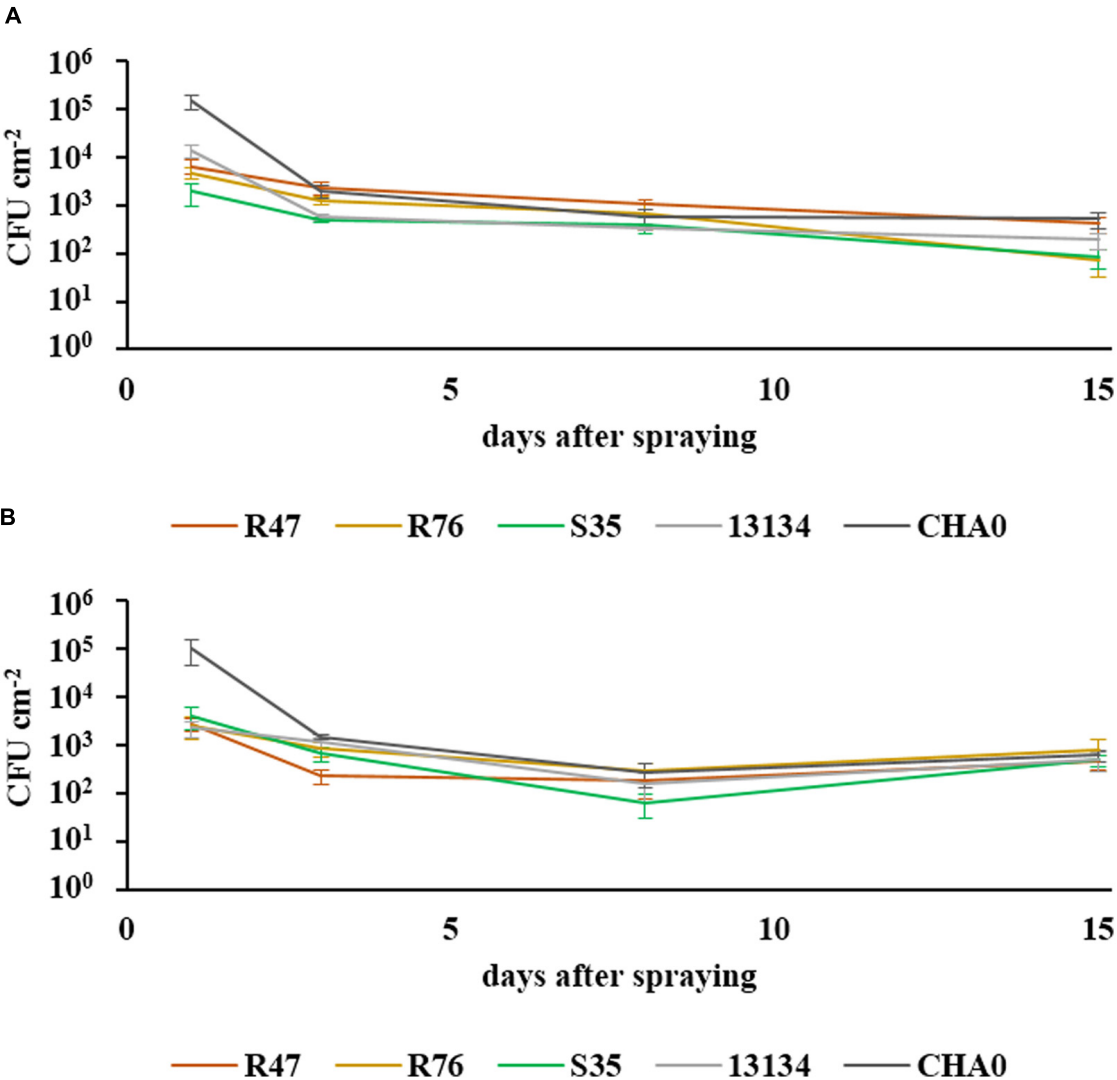
**Survival of bacteria in the phyllosphere.** The survival of sprayed bacteria on the leaf surface was measured over a period of 15 days in the cultivars Charlotte **(A)** and Victoria **(B)**. Their abundance at defined intervals is shown in CFUs cm^-2^ (SEM, *n* = 6). One-way ANOVA revealed significant differences between the strains after 3 days [*F*(4) = 3.92, *P* = 0.013 for cv. Charlotte and *F*(4) = 5.01, *P* = 0.004 for cv. Victoria], but none after 8 days and only a marginal one for cv. Charlotte at 15 days [*F*(4) = 2.77, *P* = 0.049].

The ability of the three selected strains to survive in the phyllosphere and in the rhizosphere was assessed in a greenhouse experiment, using rifampicin-resistant derivatives of the strains. The survival of bacteria in the phyllosphere was monitored at different intervals within a 15 day period after spraying the biocontrol agents onto the leaves of potato plants. After a strong decrease in population abundance within the first few days, the levels stayed almost constant during the second week and remained at ca. 100 CFUs per cm^2^ (**Figure 2**). All bacterial strains survived in the two tested cultivars for the entire tested period. For the survival in the rhizosphere, washed roots from 11 week-old sprout-inoculated potato plants were used. The abundance of the retrieved inoculated bacteria is shown in a semi-quantitative way in **Table 2.** In general, the number of bacterial CFUs from the root system of the cultivar Victoria was higher than that retrieved from the root system of Charlotte. All strains were able to establish in the rhizosphere and to compete with the natural potato microbiome, thus demonstrating their rhizosphere competence. The strain originally retrieved from the phyllosphere (S35) showed similar to higher colonization densities as the closely related *P. fluorescens* strain isolated from the rhizosphere (R76) (**Table 2**). However, highest colonization capacity was observed for *P. chlororaphis* R47: this strain was not only found in very high abundance in the pots where it had been inoculated, but it was also retrieved in significant amounts from neighboring pots (pots had been randomly placed in the greenhouse but in trays where they were connected through the bottom upon watering events), suggesting very high rhizosphere competence. It cannot be excluded that due to this invasion of *P. chlororaphis* R47, the other strains were restrained in their rhizosphere colonization and that the data presented in **Table 2** therefore underestimate their true colonization potential.

**Table 2:** Survival of bacteria in the rhizosphere.

		Inoculated strain	R47
cv. Charlotte	Control	-	+
	R47	++++	++++
	R76	++	++
	S35	+++	++
	13134	++	-
	CHA0	+++	-
cv. Victoria	Control	-	++
	R47	++++	++++
	R76	+++	++
	S35	+++	+++
	13134	+++	+++
	CHA0	+++	+++


*The inoculated strains (and R47, right) were retrieved from the roots of 11-week old plants of cv. Charlotte (top) and cv. Victoria (bottom). Abundances were estimated and are expressed as follows (CFU per gram root fresh weight): -, none detected, +, 10^*1*^–10^*3*^, ++, 10^*3*^–10^*4*^, +++, 10^*4*^–10^*6*^, ++++, >10^*6*^.*

### Evaluating the *In Planta* Protection Potential of the Strains Using a Leaf Disk Method

**FIGURE 3:**
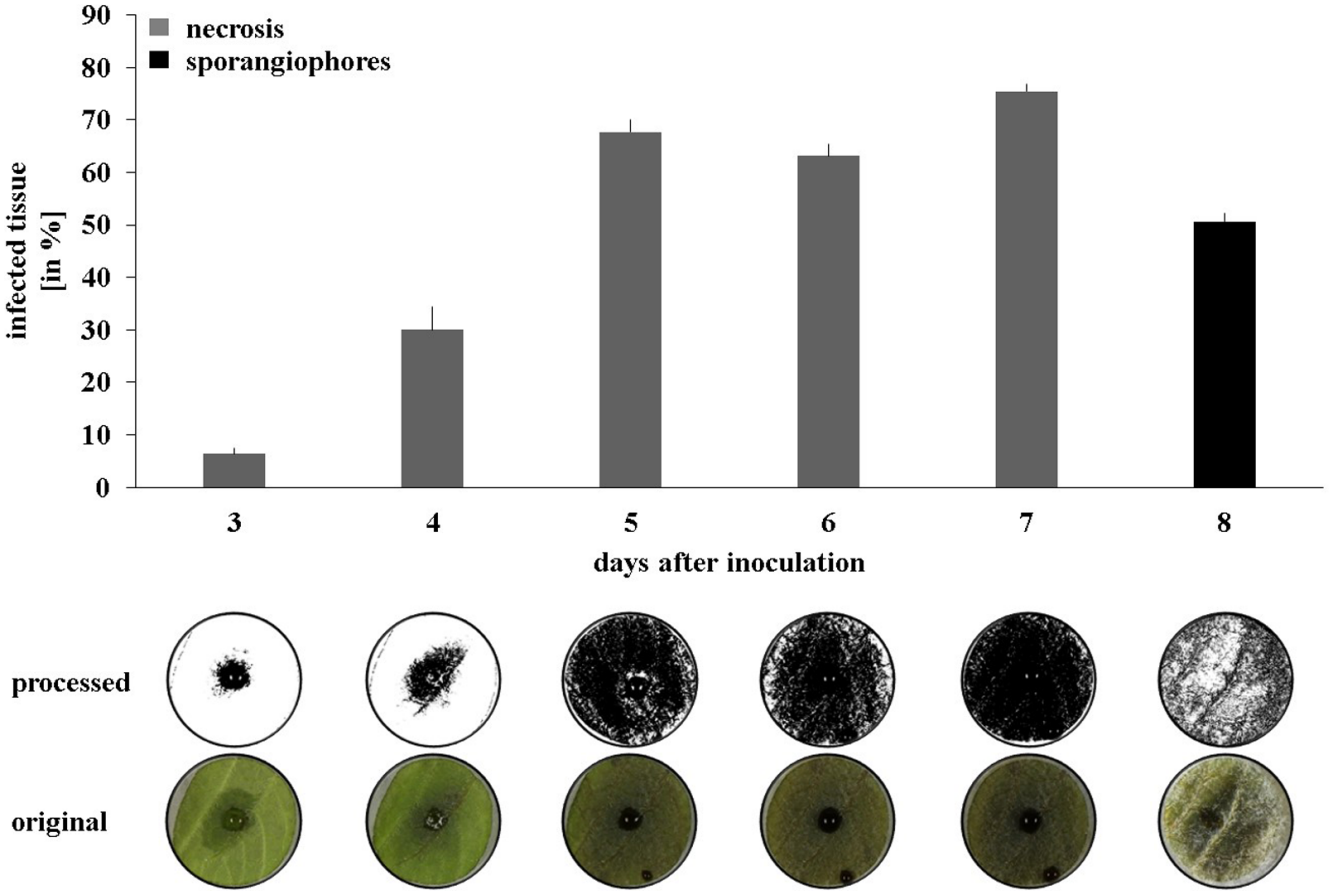
**Disease progression on potato leaf disks.** Pictures were taken daily between 2 and 8 days after inoculation and processed to quantify necrosis area (days 2–7) and sporangiophore production (day 8). Averages and standard errors are shown (*n* = 18). Pictures were taken with illumination from the top for necrosis measurement and with illumination from the side for sporangiophore measurement. One representative picture and its processed counterpart are shown per day.

As a next step toward the evaluation of the bacterial strains’ *in planta* protection potential, a high through-put leaf disk setup was developed (see Material and Methods for details). Applying the *Phytophthora* sporangia solution onto the bottom side of the leaf disk resulted in quicker disease progression than when sporangia were inoculated on the upper side (**Supplementary Figure S2**). Moreover, the drop containing the sporangia stayed in place until the end of the experiment, whereas when inoculated onto the upper side of the leaf disk, it often erratically spread and led to multiple infection starting points. To analyze the data in an automated and quantitative manner, a macroinstruction was developed in the freeware ImageJ to measure the necrosis development (days 3–7) and the formation of sporangiophores (day 8). Using a 10 μl drop of a 1.25^.^10^5^ mL^-1^ sporangia solution led to clearly distinguishable necrosis development curves and a significant covering of the leaf disk surface by sporangiophores after 8 days (**Figure 3**).

**FIGURE 4:**
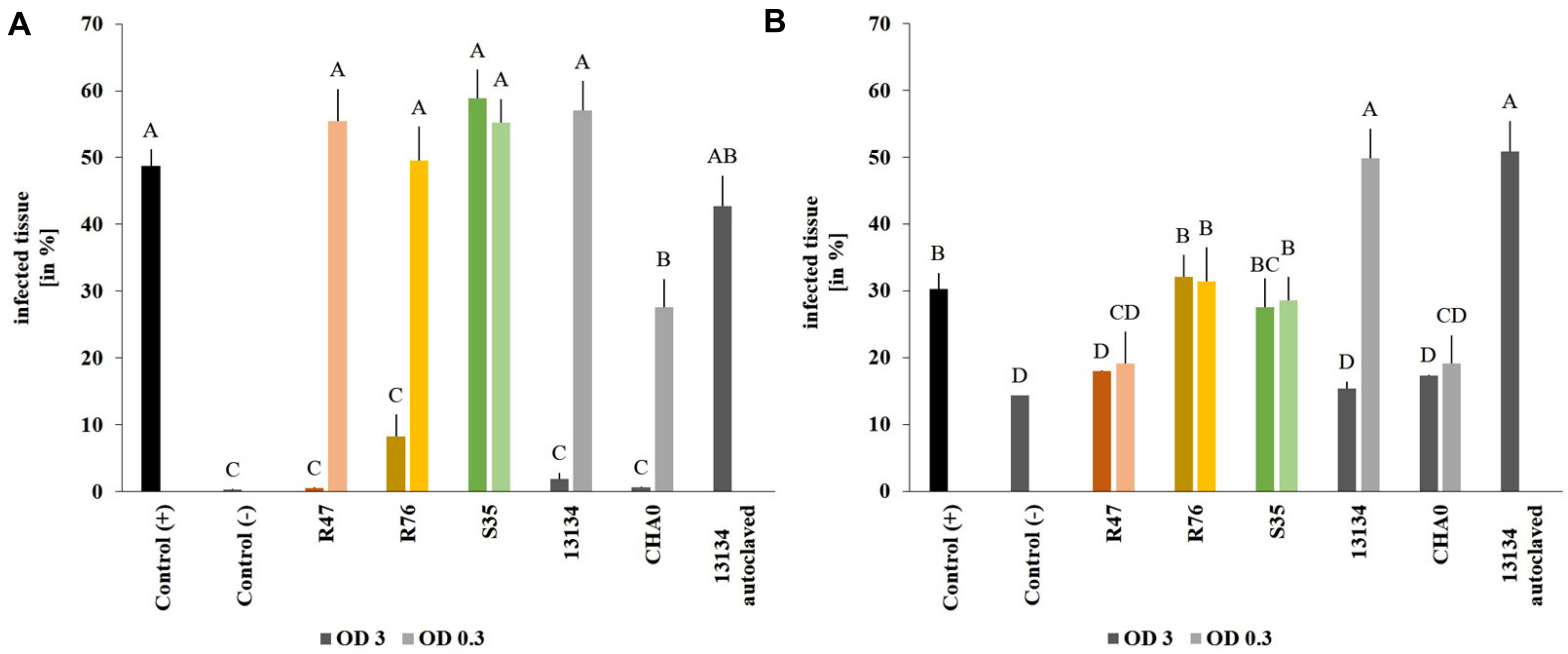
**Protective effect of the strains on leaf disks.** Sporangia and bacteria were inoculated onto leaf disks. Necrosis formation was quantified after 4 days **(A)** using a macroinstruction (see Supplementary Material, macro1) and sporangiophore production after 8 days **(B)** (macro2). OD = 3 is represented by dark and OD = 0.3 by light bars. Averages and standard errors are shown (*n* = 18–20). Different letters indicate statistically different values (Tukey’s *post hoc* test: *p* < 0.05).

Using the established leaf disk method, the effect of the three selected bacterial strains were investigated *in planta* in the greenhouse using Victoria as potato cultivar. Victoria, rather than Charlotte, was chosen for the greenhouse and field experiments due to its lesser susceptibility to late blight. Leaf disks from sprout-inoculated potato plants did not show greater tolerance to *P. infestans* than disks from non-inoculated control plants, suggesting that the inoculated strains did not induce resistance (data not shown). When bacteria were applied on the leaves at high concentration, all strains but *P. marginalis* S35 inhibited the formation of *Phytophthora-*induced necrotic lesions (**Figure 4A**). However, when a tenfold dilution of the inoculum was used, only the *P. protegens* CHA0 strain reduced necrotic area significantly, while the others did not. *P. fluorescens* R76, which strongly inhibited the formation of necrosis (8% of the leaf disk surface vs. 48% for the untreated control), was unable to reduce the formation of sporangiophores (**Figure 4B**). In contrast, *P. chlororaphis* R47 and *P. protegens* CHA0 significantly inhibited sporangiophore production in both inoculum densities tested. A very strong concentration-dependency was observed for *Pseudomonas* sp. DSMZ 13134, which conferred excellent protection when applied in high concentrations, but was inefficient or even favoring infection when applying a lower concentration. Autoclaved *Pseudomonas* sp. DSMZ 13134 cells did not confer any protection, suggesting that living bacteria are required for plant protection against *P. infestans.*

### Inhibition of Sporangia Germination by the Bacterial Strains

**FIGURE 5:**
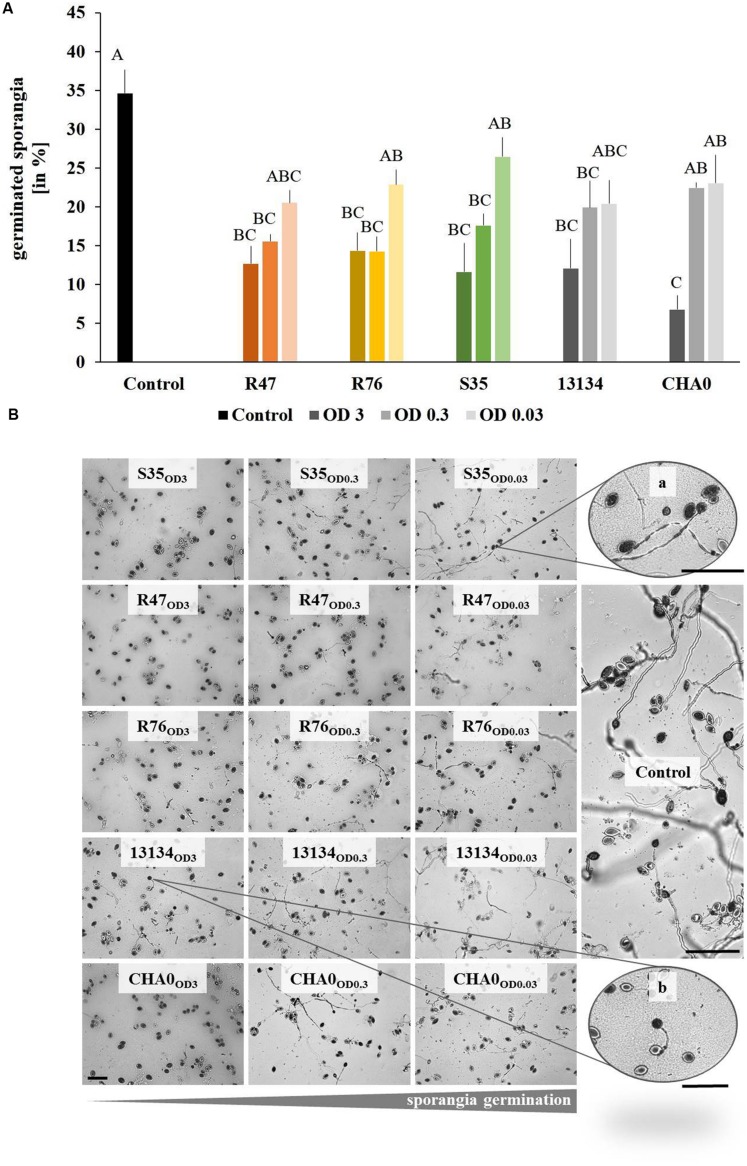
**Germination of *Phytophthora infestans* sporangia in the presence of bacteria.** Sporangia and bacteria in different concentrations were co-inoculated on agar plugs and the percentage of germinated sporangia was counted after 3 days. **(A)** Percentage of germinated sporangia. Different letters indicate significant differences (Tukey’s *post hoc* test: *p* < 0.05, *n* = 4). OD = 3 are represented by dark, OD = 0.3 by intermediate and OD = 0.03 by light bars. **(B)** Representative pictures of *Phytophthora infestans* sporangia germinated in the presence of different concentrations of bacteria. From left to right, decreasing bacteria concentration and increasing sporangia germination. Abnormal morphology such as hyphal swelling was observed even upon exposure to low bacteria concentrations (zoomed picture on the top right). Bars indicate 100 μm.

The first step in *Phytophthora*’s infection process is the germination of sporangia and/or zoospores. We therefore assessed whether the bacterial strains used in the leaf disk experiments were able to inhibit this critical step. The overall germination rate in control treatments was about 35%. When applied at high densities, all strains induced a significant reduction in the percentage of germinated sporangia (**Figure 5A**). The same observation could be made with intermediate densities, except for the control strain *P. protegens* CHA0, which was not significantly different from the untreated control. Low densities of bacteria (OD = 0.03) were ineffective in reducing sporangia germination (**Figure 5A**). In addition to the reduced germination rate, morphological abnormalities such as hyphal swelling and shorter germination tubes could be observed upon treatment with the bacterial strains (**Figure 5B**). No correlation was observed between the *in vitro* effects of the strains on sporangia germination and the protection potential observed on leaf disks. However, when microscopic observations were done on the sporangia drop applied onto the leaf disks, it seemed that the strain S35, which was unable to protect leaf disks against *P. infestans*, also did not inhibit sporangia germination as drastically as the other strains (**Supplementary Figure S3**).

### Survival and Protective Potential of the Strains in Field Conditions

**FIGURE 6:**
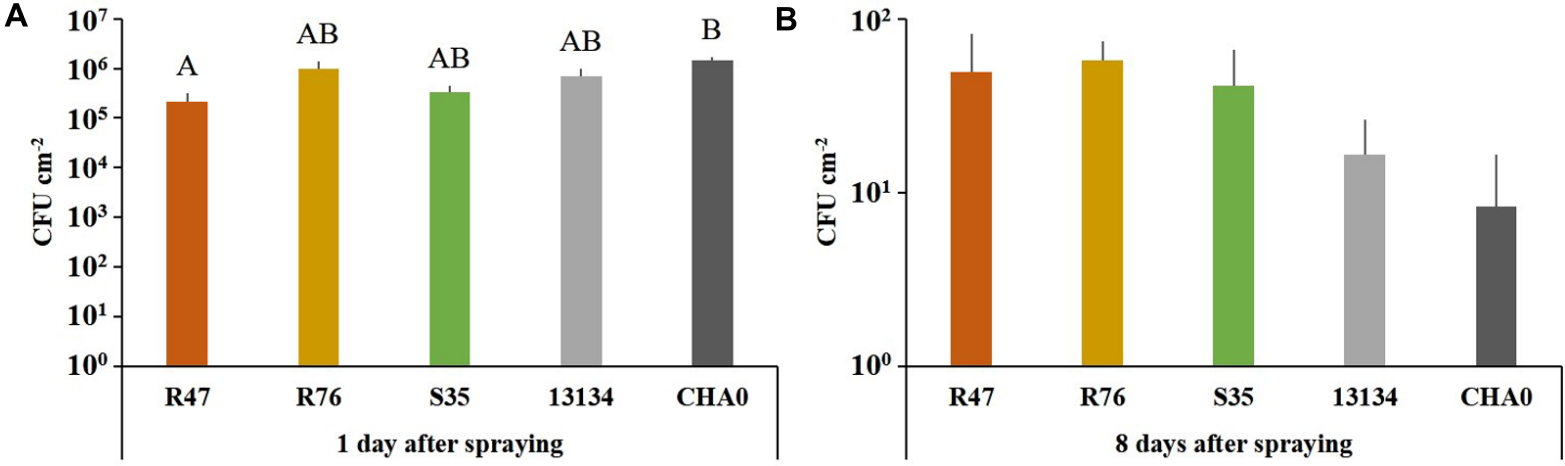
**Survival of the inoculated bacterial strains in a field microplot experiment.** Abundance of bacteria were determined on leaf disks (*n* = 4) of sprayed plants collected 1 **(A)** and 8 **(B)** days after spraying. The number of colonies after 8 days did not differ significantly between the treatments according to one-way ANOVA [*F*(4) = 1.13, *P* = 0.38], even though 1 day after spraying significant differences were observed. (Tukey’s *post hoc* test: *p* < 0.05, *n* = 4).

**FIGURE 7:**
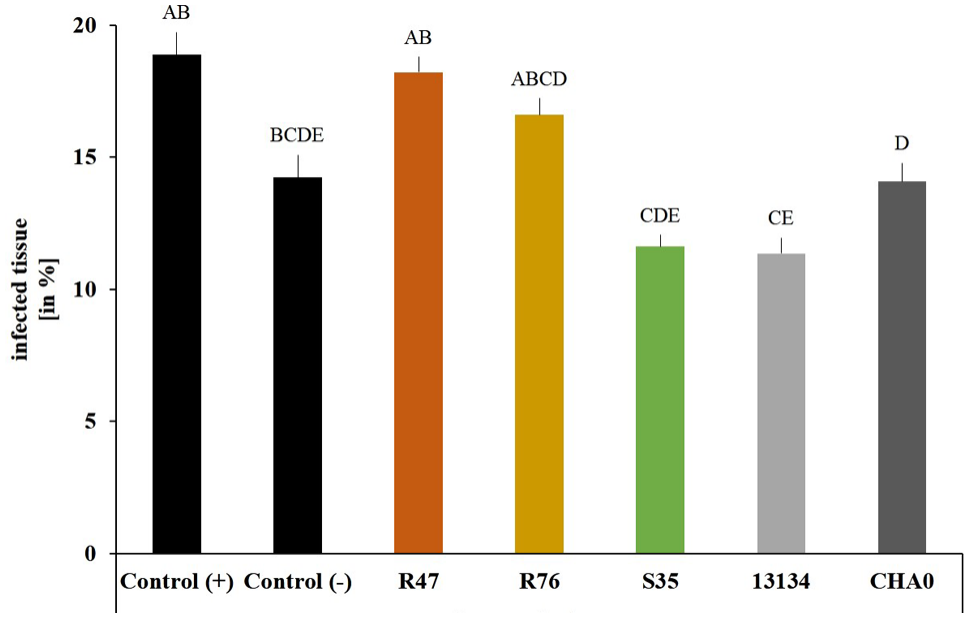
**Protective effect of the strains on leaf disks of field-grown plants.** *Phytophthora infestans* sporangia and bacteria were inoculated onto leaf disks collected from field-grown plants 1 day after spraying. Control (+) are leaf disks from untreated plants infected with *P. infestans* sporangia. Control (-) are leaf disks from untreated plants not infected with sporangia. Sporangiophore production was quantified after 6 days using a macroinstruction (see Supplementary Material, macro3). Averages and standard errors are shown (*n* = 52–58, *n*_control(-)_ = 14). Different letters indicate statistically different values (Tukey’s *post hoc* test: *p* < 0.05).

To assess whether our selected strains would be able to protect potato against *P. infestans* under field conditions, we carried out a microplot experiment where potato plants were regularly sprayed with a suspension of the bacterial strains. After the last application, both survival and protection potential were assessed. One day after spraying the bacterial suspensions on the plants, all strains were still present in relatively high abundance (10^5^–10^6^ cm^-2^) (**Figure 6**), but their population density dropped within the next days: after 8 days, only few cells per square centimeter of leaf could be retrieved. The microplot experiment was carried out to monitor the protective effects of the strains in field conditions, i.e., with natural *P. infestans* infection. Since this natural infection was prevented by very hot and dry weather conditions during July and August, we tested the protective effect of the strains with our leaf disk experimental setup. This revealed that the leaf disk method was, at least in our experiments, better suited for the greenhouse screen, since the infection was much less efficient in field-grown plants (**Figure 7**). The infected and the non-infected controls did not differ significantly from each other, which was mostly due to a lesser infection rate of the infected controls (see also **Figure 4**). Therefore, even though significant protective effects were observed after treatment with the strain *P. moraviensis* S35 as well as the two control strains *Pseudomonas* sp. DSMZ 13134 and *P. protegens* CHA0, these results should be interpreted with caution.

## Discussion

Using microorganisms as biocontrol agents seems an appealing strategy for sustainable crop protection: in the last decades, much effort has been made to isolate, characterize and use microbial strains to this end, yet the bacterial antagonists available on the market are so far only few (Velivelli et al., 2014). Indeed, many criteria have to be fulfilled for such a biocontrol agent to find its way to the farmer (Köhl et al., 2011). The first step usually taken to select for candidate biocontrol agents is an *in vitro* screening in the laboratory, such as the one we carried out against potato pathogens in the present study (**Table 1**). Compared to the first screen of the potato-associated strains reported in (Hunziker et al., 2015), which focused on volatiles, the present evaluation of the strains’ potential activity against a broad range of potato pathogens yielded slightly different results: while the *Pseudomonas* strains classified as the best producers of antifungal volatiles (Hunziker et al., 2015) also were very active in the present study, other, non-*Pseudomonas* strains such as the two *Bacillus* strains R73 and R54 or the *Streptomyces* S01 were much more inhibitory to the potato pathogens when their diffusible substances, rather than only their volatiles, came into play. Moreover, the selective inhibition of *Helminthosporium solani* by strains affiliated with the species *P. frederiksbergensis* seem to originate from diffusible substances, as these strains did not produce volatile compounds inhibiting *H. solani* (Hunziker et al., 2015).

However, the results obtained in such *in vitro* experimental setups only represent a metabolic potential of the strains grown on rather rich laboratory media, and no guarantee can be offered that the strains that are active *in vitro* will also be *in planta.* The reasons for this first screen in the laboratory are mainly time and space constraints. Therefore, developing a space- and time-efficient screening procedure, which would allow testing the biocontrol agents at a very early stage already on plant material rather than on artificial laboratory media would potentially yield better-suited candidate strains for further investigations. This is why we developed a high through-put leaf disk method allows the monitoring over time and the automated quantification of disease progression by picture analysis. The main difficulty in developing a reliable macroinstruction to quantify the sporangiophore production came from the white background originating from leaf veins and trichomes. Nevertheless, provided the macroinstructions are carefully adapted to each new experimental setup, the automated leaf disk quantification method represents a big step forward for the selection of promising biocontrol strains, since it provides the means to obtain in a time- and space-efficient manner quantitative data on the *in planta* disease protection potential of the strains of interest. While this assay was in our hands well-suited for greenhouse-grown plants, it was not suitable to evaluate disease progression on material from field-grown plants, since the control plants (of the same cultivar, Victoria) developed little infection (less than 20% of leaf surface infected, compared with over 30% for leaf disks from greenhouse plants, **Figures 4** and **7**). Field-grown plants are expected to show a basic level of resistance to diseases due to the multifarious biotic and abiotic stimuli they encounter in nature (Walters, 2009). Moreover, plants grown in the greenhouse show less abundant and less diverse microbial colonization than field-grown plants (data not shown), which might also explain their higher susceptibility to *P. infestans* infection, as the role of the plant microbiome in disease protection and induction of resistance is becoming increasingly clear (Bakker et al., 2013; Pieterse et al., 2014). The leaf disk method and the subsequent automated image analysis developed in the present study is therefore not meant to replace whole-plant analysis and field trials, but represents an efficient tool to replace the *in vitro* screening and to select for antagonists that are able to inhibit the pathogen when both organisms grow on the host plant rather than on rich laboratory media. Since the co-inoculation of both antagonist and pathogen would not allow to see induced effects, the setup might be adapted by either applying the antagonists 1 day before the pathogen or by spraying whole plants with the antagonists and thereafter cutting leaf disks, infecting them with *P. infestans* and monitoring disease progression.

In our case, the strain that showed the highest *in vitro* activity turned out to also be the most efficient when tested on leaf disks from greenhouse-grown plants (**Table 1**, **Figure 4**), although the very few strains selected in this study do not allow any generalization of this observation. This *Pseudomonas* strain was affiliated to the *P. chlororaphis* species according to its *rpoD* sequence (Hunziker et al., 2015), a species to which the active ingredient of the product Cerall^®^ also belongs (Johnsson et al., 1998; Velivelli et al., 2014). This affiliation was confirmed by phylogenetic analysis based on four housekeeping genes (*16S rRNA*, *gyrB*, *rpoB*, *rpoD*), which placed this strain in a cluster comprised of *P. chlororaphis* and *P. protegens* strains (De Vrieze et al., 2015). Both species are part of the larger *P. fluorescens* group and recent phylogenetic studies indicate a close proximity between *P. chlororaphis* and *P. protegens* (Gomila et al., 2015). Strains belonging to both species include well-known biocontrol agents against pathogenic fungi (Haas and Defago, 2005) but also against insects (Kupferschmied et al., 2013; Ruffner et al., 2013). Preliminary inspection of the genomic potential of *P. chlororaphis* R47 revealed that this strain shows a similar toolset of antibiotics as other *P. chlororaphis* strains according to a recent study comparing *Pseudomonas* strains (Loper et al., 2012): the genome of *P. chlororaphis* R47 encodes the synthesis of the antibiotics hydrogen cyanide, phenazines, pyrollnitrin and 2-hexyl-5-propyl-alkylresorcinol (HPR) (data not shown), which might be involved in its anti-*Phytophthora* activity. Moreover, siderophore (pyoverdine, achromobactin) production is also encoded in the genome and might contribute to the strains’ antifungal activity and, perhaps more importantly, to its remarkable rhizosphere competence (Ghirardi et al., 2012). Indeed, *P. chlororaphis* R47 strain seemed to largely surpass the other strains tested in terms of rhizosphere colonization (**Table 2**), an important feature considering the practical advantages (feasibility and cost-efficiency) of tuber treatment compared with leaf spraying. However, to successfully inhibit late blight at the shoot level, the biocontrol agent should be able to either induce resistance or to systematically colonize the upper parts of the plants. Although induction of resistance in potato has been shown for other *Pseudomonas* strains (Arseneault et al., 2014; Pieterse et al., 2014), leaf disks from *P. chlororaphis* R47- tuber inoculated plants did not show increased resistance to late blight (data not shown), suggesting that this strain was not able to induce long-lasting resistance to late blight after tuber inoculation. Preliminary data suggest that *P. chlororaphis* R47 might be able to move from the tuber to the upper parts of the plants, which would be an important feature for late blight protection. Such an endophytic colonization has been shown for other plant-growth promoting and biocontrol *Pseudomonas*, such as *P. poae* in sugar beet (Zachow et al., 2015) or *P. putida* in potato (Andreote et al., 2009).

For non-endophytic microbes, the challenge of a successful application as leaf spray is particularly high in view of the harsh conditions that prevail in the phyllosphere, such as UV irradiation, as well as extreme variations in temperature and humidity. In our greenhouse experiments, all tested bacterial strains were able to persist for 2 weeks, but in the field, their abundance underwent a more rapid decrease, although all inoculated strains could be retrieved after 8 days in our microplot experiment (**Figure 6**). In addition to the abiotic stresses prevailing in field conditions, the higher complexity of the microbiome in field-grown plants is also likely to reduce the ability of introduced bacteria to establish in leaves as well as in roots, due to the higher competition they are facing. The colonization ability of the strains might thus depend on the residing microbiome as well as on the plant variety: in our root colonization assay, consistently more bacteria could be retrieved from the rhizosphere of cv. Victoria than from that of cv. Charlotte (**Table 2**).

As in many studies preceding the current one, going from the greenhouse to the field proved a challenging step. However, we selected only few strains that had been pre-screened and characterized based on *in vitro* experiments. We hope that the leaf disk-based automated picture analysis of disease progression developed in the frame of this study will enable to skip this first time-consuming step of *in vitro* tests and to directly test the antagonists’ protective potential on plant material from different potato cultivars differing in their susceptibility to late blight. In a second step, the selected strains should be tested for their ability to establish sufficient population densities on field-grown plants that harbor their own, complex native microbiome, since colonization of these plants might be more challenging than that of greenhouse-grown plants harboring less complex microbial communities. Beneficial or antagonistic interactions between the inoculated strain and the resident microbiome, whose composition will change according to biotic and abiotic factors, might be important factors that support or prevent successful establishment of a biocontrol agent. Those strains showing *in planta* anti-oomycete activity in a broad range of cultivars, as well as the ability to establish on already colonized plants would then represent better candidates for the time-consuming field trials than those selected solely based on *in vitro* tests in Petri dishes. Finally, future research will tell whether the best use of microbial control agents to limit late blight in tomorrow’s potato production will reside in application of single, highly potent strains, or of a combination of strains with different and therefore complementary abilities, or in a more global approach involving microbiome engineering and microbiome-driven selection (Mueller and Sachs, 2015).

## Author Contributions

AG, LW, and AB designed the research, AG, MV, DB, RG, and NB performed experiments, AG, MV, and AB analyzed the data, LW and AG wrote the MS with help from MV, AB, NB, TM, and RG.

## Conflict of Interest Statement

The authors declare that the research was conducted in the absence of any commercial or financial relationships that could be construed as a potential conflict of interest.
