# Supplementary material for: The Anti-Phytophthora Effect of Selected Potato-Associated Pseudomonas Strains: From the Laboratory to the Field
Source: Front Microbiol. 2015 Nov 27;6:1309. doi: 10.3389/fmicb.2015.01309 (PMC4661289; doi:10.3389/fmicb.2015.01309)
Supplement: Supplementary file 1 [file Data_Sheet_1.PDF]

Supplementary Material

Figure S1 Effect of sprout inoculation on plant development (A,B) and height (C,D). This experiment was carried out with two cultivars, Charlotte (A,C) and Victoria (B,D). The development stage according to the BBCH scale as well as the height were recorded weekly. Averages of six replicates and standard errors are shown.

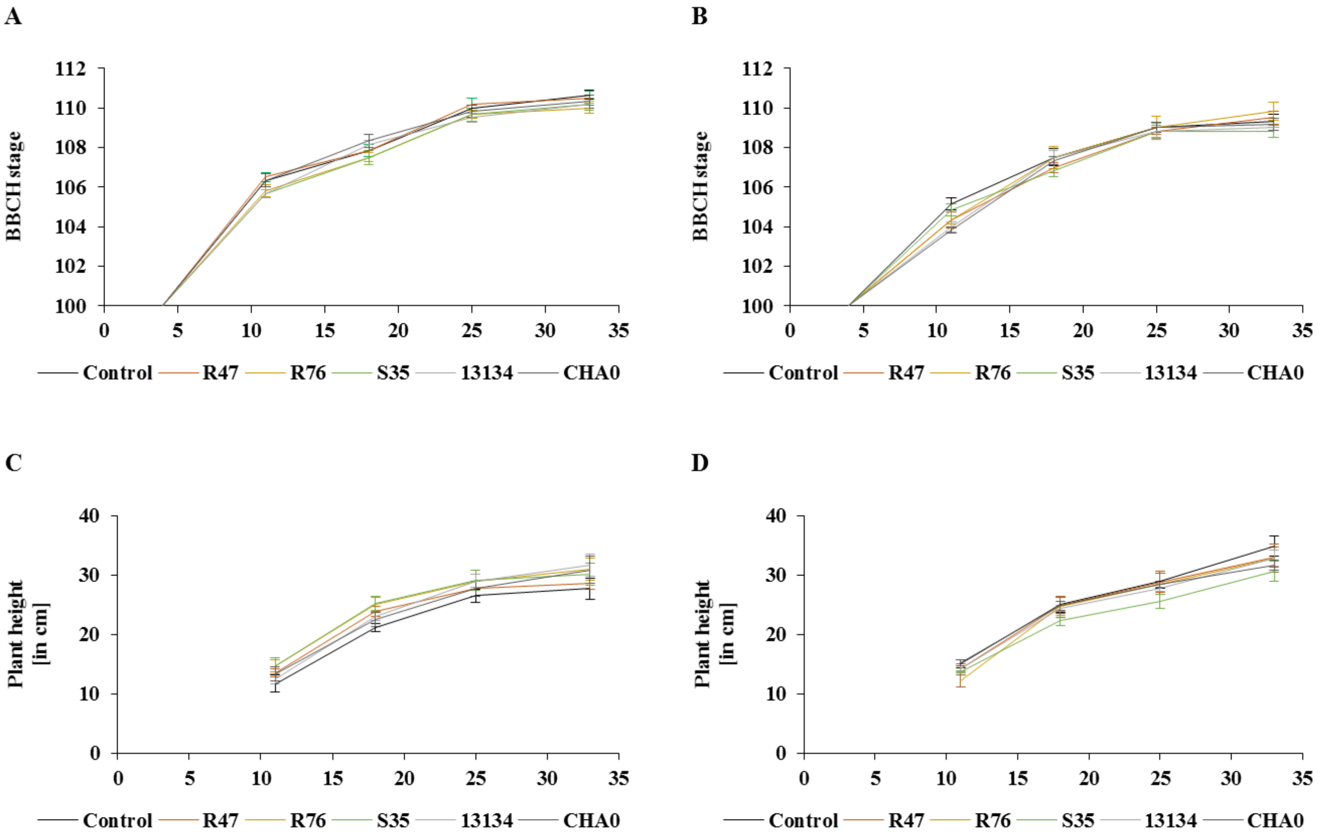

Figure S2: Setup of the leaf disc method. Sporangia were inoculated either on the upper (A) or on the lower (B) side of the leaf and disease progression was monitored daily between 3 and 8 days after inoculation. Different sporangia concentrations were used: ■, 62’500; ♦, 125’000, ▲, 250’000, and ●, 500’000. Averages of 18 replicates and standard errors are shown.

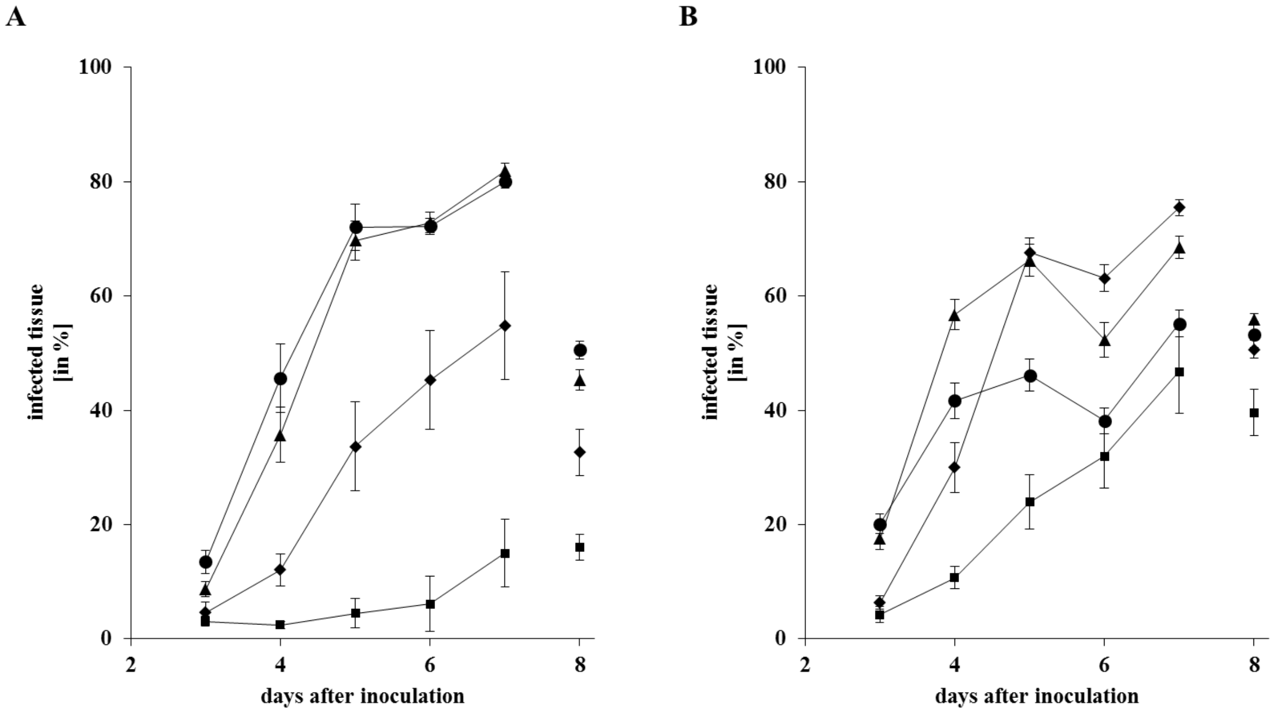

Figure S3: Representative pictures of sporangia germinated on the leaf discs under exposure to the three different strains. Picture were taken four days after inoculation.

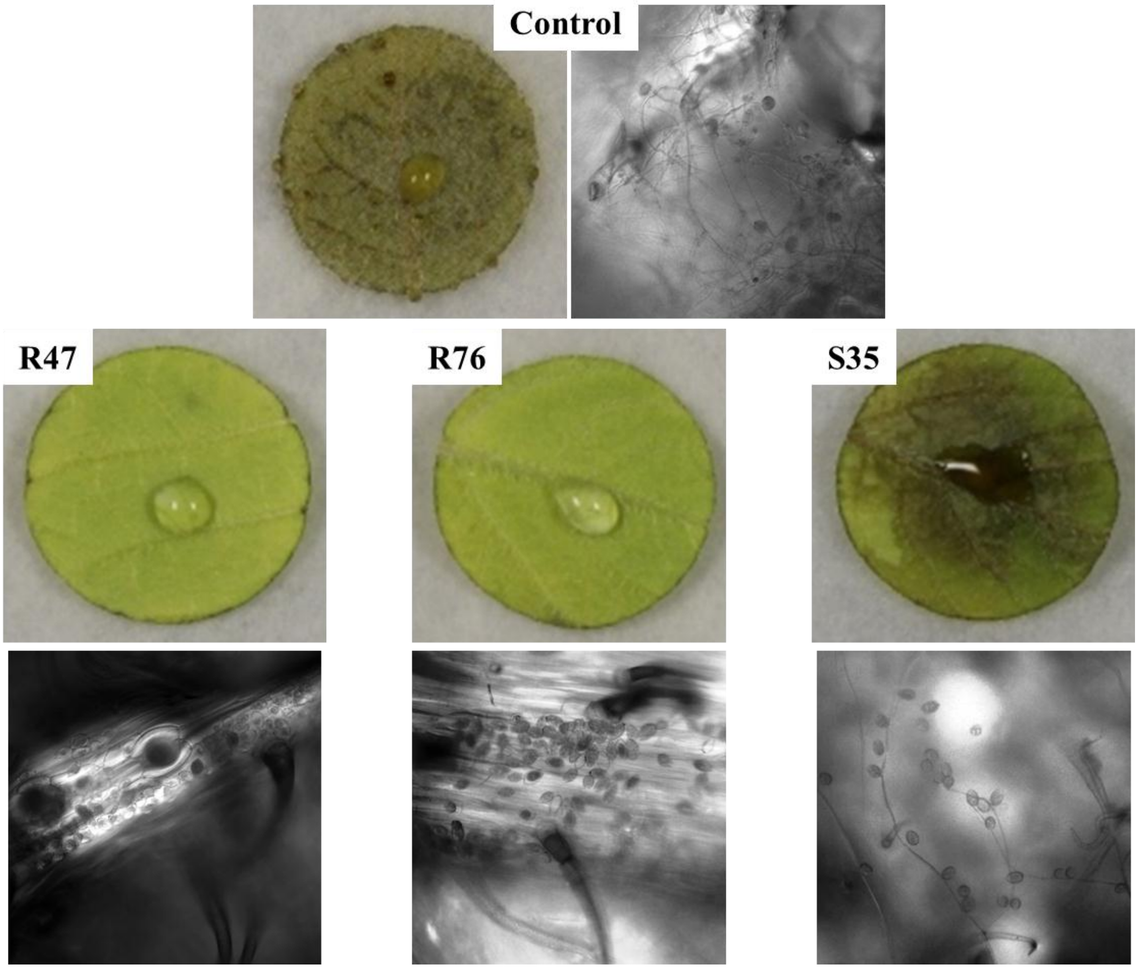

For macroinstructions, see separate text files:

macro1, macroinstruction used to measure necrosis on leaf discs (greenhouse)

macro2, macroinstruction used to measure sporangiophores on leaf discs (greenhouse)

macro3, macroinstruction used to measure sporangiophores on leaf discs (microplot)
