# Supplementary material for: The Anti-Phytophthora Effect of Selected Potato-Associated Pseudomonas Strains: From the Laboratory to the Field
Source: Front Microbiol. 2015 Nov 27;6:1309. doi: 10.3389/fmicb.2015.01309 (PMC4661289; doi:10.3389/fmicb.2015.01309)
Supplement: Supplementary file 3 [file Data_Sheet_3.DOCX]

//(c)Aurelien Bailly - Agroscope, Switzerland

run("Colors...", "foreground=white background=black selection=red");

dir = getDirectory("Choose input Directory ");

output = getDirectory("Choose output Directory ");

results = getDirectory("Choose results Directory ");

list=getFileList(dir);

for(i=0;i<list.length;i++){

open(dir+list[i]);

Loop();

}

selectWindow("Summary")

saveAs("Text", results+'Summary.xls');

print("Done!");

function Loop() {

name = getTitle();

DilateSplits();

wait(200);

FindDiscs();

selectWindow("Result of step1 (green)");

run("Close");

selectWindow("step2");

roiManager("open", output+name+'-ROIset.zip');

roiManagerSelect();

selectWindow("step2");

run("Close");

selectWindow("ROI Manager");

run("Close");

selectWindow("W&L");

run("Close");

}

function DilateSplits() {

selectWindow(name);

run("Duplicate...", "title=step1");

selectWindow(name);

run("Duplicate...", "title=step2");

selectWindow(name);

run("Close");

selectWindow("step1");

run("Split Channels");

wait(200);

selectWindow("step1 (red)");

run("Close");

selectWindow("step1 (green)");

run("Maximum...", "radius=5");

imageCalculator("Difference create", "step1 (green)","step1 (blue)");

selectWindow("step1 (blue)");

run("Close");

selectWindow("step1 (green)");

run("Close");

selectWindow("Result of step1 (green)");

run("Window/Level...");

setMinAndMax(99, 101);

run("Apply LUT");

run("Maximum...", "radius=2");

run("Make Binary");

run("Close-");

run("Dilate");

run("Close-");

run("Dilate");

run("Close-");

run("Dilate");

run("Close-");

run("Dilate");

run("Close-");

run("Dilate");

}

function FindDiscs() {

run("Analyze Particles...", "size=200000-400000 circularity=0.1-1.00 show=Nothing add");

roiManager("Show All with labels");

roiManager("Show All");

roiManager("deselect");

roiManager("save", output+name+'-ROIset.zip');

roiManager("delete");

}

function roiManagerSelect() {

n = roiManager("count");

for (j=0; j<n; j++) {

roiManager("select", j);

run("Fit Circle");

run("Copy");

run("Internal Clipboard");

selectWindow("Clipboard");

rename(name+'Disc_'+j);

selectWindow(name+'Disc_'+j);

run("Unsharp Mask...", "radius=1 mask=0.80");

run("Subtract Background...", "rolling=10 disable");

run("Invert");

run("8-bit");

setAutoThreshold("Default");

//run("Threshold...");

setThreshold(0, 190);

run("Convert to Mask");

width = getWidth();

height = getHeight();

makeOval(10, 10, width-20, height-20);

run("Analyze Particles...", "size=0-Infinity circularity=0.00-1.00 show=Nothing summarize");

selectWindow(name+'Disc_'+j);

saveAs("tiff", output+name+'_Disc_'+j);

run("Close");

}

}
